# Replication Timing: A Fingerprint for Cell Identity and Pluripotency

**DOI:** 10.1371/journal.pcbi.1002225

**Published:** 2011-10-20

**Authors:** Tyrone Ryba, Ichiro Hiratani, Takayo Sasaki, Dana Battaglia, Michael Kulik, Jinfeng Zhang, Stephen Dalton, David M. Gilbert

**Affiliations:** 1 Department of Biological Science, Florida State University, Tallahassee, Florida, United States of America; 2 Department of Biochemistry and Molecular Biology, University of Georgia, Athens, Georgia, United States of America; 3 Department of Statistics, Florida State University, Tallahassee, Florida, United States of America; MRC Laboratory of Molecular Biology, United Kingdom

## Abstract

Many types of epigenetic profiling have been used to classify stem cells, stages of cellular differentiation, and cancer subtypes. Existing methods focus on local chromatin features such as DNA methylation and histone modifications that require extensive analysis for genome-wide coverage. Replication timing has emerged as a highly stable cell type-specific epigenetic feature that is regulated at the megabase-level and is easily and comprehensively analyzed genome-wide. Here, we describe a cell classification method using 67 individual replication profiles from 34 mouse and human cell lines and stem cell-derived tissues, including new data for mesendoderm, definitive endoderm, mesoderm and smooth muscle. Using a Monte-Carlo approach for selecting features of replication profiles conserved in each cell type, we identify “replication timing fingerprints” unique to each cell type and apply a k nearest neighbor approach to predict known and unknown cell types. Our method correctly classifies 67/67 independent replication-timing profiles, including those derived from closely related intermediate stages. We also apply this method to derive fingerprints for pluripotency in human and mouse cells. Interestingly, the mouse pluripotency fingerprint overlaps almost completely with previously identified genomic segments that switch from early to late replication as pluripotency is lost. Thereafter, replication timing and transcription within these regions become difficult to reprogram back to pluripotency, suggesting these regions highlight an epigenetic barrier to reprogramming. In addition, the major histone cluster Hist1 consistently becomes later replicating in committed cell types, and several histone H1 genes in this cluster are downregulated during differentiation, suggesting a possible instrument for the chromatin compaction observed during differentiation. Finally, we demonstrate that unknown samples can be classified independently using site-specific PCR against fingerprint regions. In sum, replication fingerprints provide a comprehensive means for cell characterization and are a promising tool for identifying regions with cell type-specific organization.

## Author Summary

While continued advances in stem cell and cancer biology have uncovered a growing list of clinical applications for stem cell technology, errors in indentifying cell lines have undermined a number of recent studies, highlighting a growing need for improvements in cell typing methods for both basic biological and clinical applications of stem cells. Induced pluripotent stem cells (iPSCs)—adult cells reprogrammed to a pluripotent state—show great promise for patient-specific stem cell treatments, but more efficient derivation of iPSCs depends on a more comprehensive understanding of pluripotency. Here, we describe a method to identify sets of regions that replicate at unique times in any given cell type (replication timing fingerprints) using pluripotent stem cells as an example, and show that genes in the pluripotency fingerprint belong to a class previously shown to be resistant to reprogramming in iPSCs, identifying potential new target genes for more efficient iPSC production. We propose that the order in which DNA is replicated (replication timing) provides a novel means for classifying cell types, and can reveal cell type specific features of genome organization.

## Introduction

In mammals, replication of the genome occurs in large, coordinately firing regions called replication domains [1]–[7]. These domains are typically one to several megabases, roughly align to genomic features such as isochores, and are closely tied to subnuclear position, with transitions to the nuclear interior often coupled to earlier replication, and transitions to the periphery to later replication [4], [5], [8], [9]. Given their connections to subnuclear position and remarkably strong correlation to chromatin interaction maps [3], replication profiles provide a window into large-scale genome organization changes important for establishing cellular identity. The organization of replication domains is cell-type specific, and a larger number of smaller replication domains is a property of embryonic stem cells (ESCs) [3]–[5]. Importantly, in both humans and mice, induced pluripotent stem cells (iPSCs) reprogrammed from fibroblasts display a timing profile almost indistinguishable from ESCs, suggesting that replication profiles may also be used to measure cellular potency [3], [5].

While a wide-range of cell classification methods are actively used, the most common practice for verifying identity is to monitor a handful of molecular markers, some of which are shared with other cell types. Genome-wide classification of features such as DNA methylation [10]–[12], transcription [13], [14] and histone modifications [15], [16] have in principle more potential to accurately distinguish specific cell types. However, these features of chromatin are highly dynamic at any given genomic site [17], and most measurements require high-resolution arrays and costly antibodies. Moreover, recent reports highlight the unstable nature of transcription and related epigenetic marks in multiple embryonic stem cell lines [18], [19]. By contrast, since replication is regulated at the level of large domains, replication profiles are considerably less complex to generate and interpret than other molecular profiles. Timing changes occurring during differentiation are on the order of several hundred kilobases and are highly reproducible between various stem cell lines [3]–[5]. They are also robust to changes in individual chromatin modifications, retaining their normal developmental pattern in G9a(−/−) cells despite strong upregulation of G9a target genes and near-complete loss of H3K9me2 [8].

Here, we describe a method for classifying cell types—replication fingerprinting—based on genome-wide replication timing patterns in mouse and human ESCs and other cell types. We applied the method to 67 (36 mouse and 31 human) whole-genome replication timing datasets to demonstrate the feasibility of classifying cell types using a minimal set of cell type-specific regions. After identification, these regions were used to classify two independent samples using site-specific PCR. We also demonstrate that loss of pluripotency is accompanied by consistent changes in replication timing, implicating the replication program as an important factor in maintaining pluripotency and revealing a novel fingerprint for pluripotent stem cells.

## Results

### Generation of replication profiles

**Figure 1:**
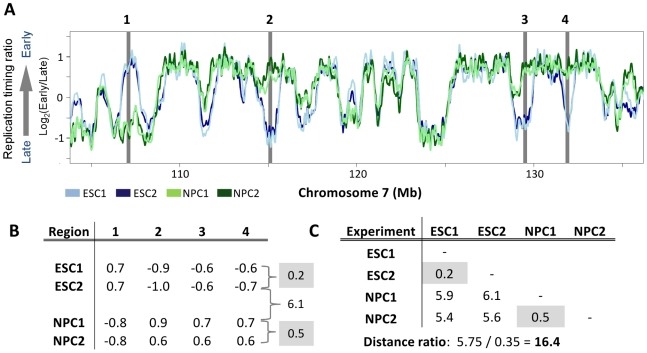
A simplified replication timing fingerprint. **A.** Four 200 kb regions in chromosome 7, highlighted in grey, are selected for a simplified fingerprint using two replicates each of ESCs (light and dark blue) and NPCs (light and dark green). **B.** The replication timing ratio for each region in each experiment is shown, with the total distances in replication timing for all fingerprinting regions between replicates of ESCs or NPCs in grey. Note that distances between the two different cell types (ESC vs. NPC) are substantially higher than those between replicate profiles (e.g., 6.1 for ESC2 vs. NPC1; shown between the grey boxes). **C.** Total differences in replication timing for all four fingerprinting regions between all combinations of the two replicates from these two cell types are shown. Highlighted in grey are the values for the two replicates of each cell type, which are considerably less than the values for any of the inter-cell type comparisons. Shown below the table is the “Distance ratio”, calculated as the average distance between cell types (or between replicates) divided by the average distance within cell types. The Distance ratio represents the degree of separation between replication profiles in regions used for classification.

In addition to our previously reported replication profiles, BG02 hESCs were differentiated to mesendoderm and definitive endoderm as previously described [20], as well as ISL+ mesoderm and smooth muscle cultured in defined medium (Methods), and profiled for replication. Replication profiles were generated as described previously [3]–[5], [21]. In brief, nascent DNA fractions were collected in early and late S-phase, differentially labeled, and co-hybridized to a whole-genome CGH microarray. The ratio of early and late fraction abundance for each probe—“replication timing ratio”—represents its relative time of replication. Values from individual probes are then smoothed using LOESS (a locally weighted smoothing function), and plotted on log scale (Figure 1). Replication profiles generated in this way are freely available to view or download at www.ReplicationDomain.org [22], and those analyzed in this report are summarized in Table S1.

### Generation of replication fingerprints

Figure 1 illustrates the basic concept of replication fingerprinting. Two exemplary profiles each for D3 embryonic stem cells (ESCs; blue) and D3 ESC-derived neural precursor cells (NPCs; green) are overlaid. Given that most of the genome is conserved in replication timing between any two cell types (e.g. 80% conserved between ESCs and NPCs [4]), the first challenge is to choose genomic regions that are differentially replicated within a set of cell types. We define a “replication fingerprint” of a cell type as a set of genomic regions useful for classification, along with their associated replication timing values. For a simplified example, we show exemplary fingerprint regions for a segment of chromosome 7 (Figure 1A, gray bars). Note that the four regions change dramatically upon differentiation to neural precursors (e.g., ESC2 vs. NPC1; Figure 1A,B), but have replication timing values that are well conserved between replicate experiments (e.g., ESC1 vs. ESC2). We and others have observed similarly widespread changes in replication profiles between any two different cell types profiled to date [1], [3]–[5], [7].

As classification methods require a measure of distance between samples, we defined the distance between replication profiles as the sum of absolute differences in replication timing in fingerprinting regions (Figure 1B). To select an optimal set of fingerprinting regions we maximize a “distance ratio,” representing the ratio of the average distance between unlike cell types to the average distance between equivalent cell types (Figure 1C). This ratio is maximized by selecting regions that are consistently different in replication timing between different cell types, but consistently similar between equivalent types. Importantly, the assignment of unlike vs. equivalent cell types is user-defined and flexible, allowing selection of features that best distinguish any group of cells from any other, such as ESCs from NPCs, normal from disease-related cells, or pluripotent from committed cells.

While Figure 1 shows a simplified example of four regions distinguishing ESCs from NPCs, real-world classification requires the ability to make distinctions genome-wide between many cell types, making manual selection of regions impractical. Therefore, to make the method generally applicable, we developed an automated algorithm based on Monte Carlo sampling [23] to select regions that best distinguish between all available cell types in genome-wide replication datasets. Alternative approaches evaluated for feature selection and classification included Bayesian networks, nearest neighbor methods, decision trees and SVMs, which were comparably successful only for smaller collections of cell types. We chose to explicitly maximize distances between cell types in the method described here in anticipation of translating cell classification to more convenient empirical assays with a limited number of features, because larger timing differences are easier to verify empirically and are more robust to experimental and biological variation.

### Monte Carlo optimization of fingerprint regions

In practice, replication fingerprinting is a feature selection problem. Although most genome-wide approaches are both simple and comprehensive, we found that genome-wide correlations and distances, while a good first approximation of the relatedness between cell types, are not ideal for classification as the small amount of noise in regions with conserved replication timing is compounded over this relatively large fraction of the genome (Figure S1). We therefore wish to exclude domains that are noisy (having high technical or biological variability), irrelevant (conserved in all cell types), or redundant (containing overlapping information). To achieve this, we first remove regions with conserved replication timing between cell types, resulting in a set of informative regions that can be further optimized by a Monte Carlo selection algorithm.

Figure S2 depicts the Monte Carlo algorithm. To reduce noise from individual probe measurements, replication profiles are first averaged into nonoverlapping windows of approximately 200 kb. This window size represents a balance between sizes of the regions that change replication timing during development (400–800 kb), and the number of probes needed for timing changes to be deemed statistically significant (35–180 probes are contained in each window depending on the probe density of the array platform; see Methods, Table S2). An initial set of regions with the highest replication timing changes in the set of replication profiles are chosen to exclude regions with conserved replication timing, and half of these starting regions are randomly selected to calculate initial distances between cell types. At each iteration of the algorithm, a region can be added to the set of fingerprint regions, removed from the set, or swapped with an unused region. Using a Metropolis-Hastings criterion [23], [24], moves that improve the overall distance ratio are accepted with higher probability than those that do not; after 20,000 or more such moves, a final set of fingerprinting regions is selected.

**Figure 2:**
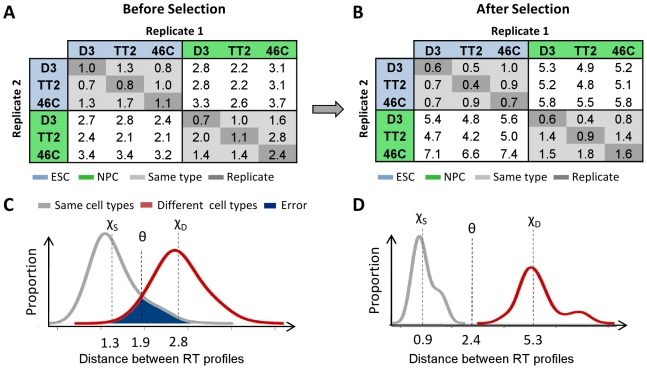
Monte Carlo optimization of fingerprinting regions. A Monte Carlo algorithm is used to select regions with maximal differences in replication timing between cell types and minimal differences between replicates to obtain an optimized set of genomic regions for classification using the nearest-neighbor method. **A,B.** Selection of fingerprinting regions accentuates differences between cell types while diminishing those within equivalent cell types (light gray) and replicates (dark gray). **C,D.** To calculate confidence levels of predictions we use the distributions of distances within (grey) and between (red) cell types, shown here for 30 runs before and after selection. The error rate of prediction is represented by the blue shaded area shared by comparisons between similar or distinct cell types, with average distances of χ_S_ and χ_D_ respectively. The optimal classifier, θ, is estimated by minimizing the number of misclassified distances as in Figure 3 and Figure 4. Above this distance, datasets are predicted to originate from different cell types.

**Figure 3:**
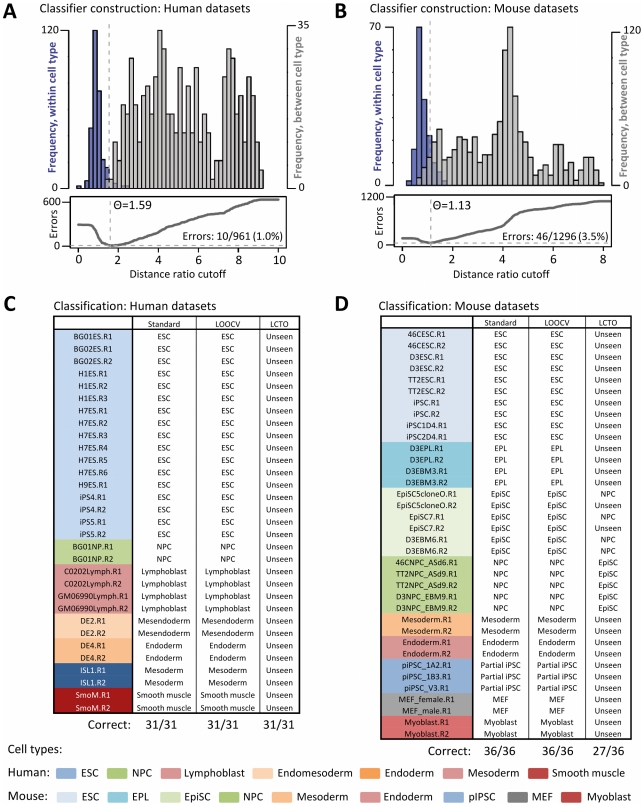
Cell type classification using Monte-Carlo selected domains. **A,B.** (Top panel) Distribution of distances within (blue) and between (gray) all human replication profiles for consensus fingerprinting domains in human (**A**) and mouse (**B**) cell types. (Bottom panel) Number of classification errors as a function of distance ratio cutoff. The optimal classifier (θ) is that which minimizes classification errors, with distances above θ hypothesized to originate from different cell types. **C,D.** Human dataset classification results for the standard kNN method (Standard) leave-one-out crossvalidation (LOOCV), and with each cell type excluded from training (LCTO). For LOOCV, each experiment (e.g., BG01ES.R1) is classified using 20 regions selected with that experiment left out. For LCTO, experiments are labeled as the most similar type in the training set, or correctly classified as “Unseen” for distances above θ. Experimental replicates are denoted with suffixes ‘R1’, ‘R2’, etc, and are described in Table S1.

As depicted in Figure 2, the fingerprinting algorithm selects domains with large and reproducible replication timing differences between cell types, discarding those with minimal or variable changes. Before selecting optimal regions (Figure 2A,C), the average distance between “like” and “unlike” cell types are similar, translating into classification errors for randomly selected domains (Figure 2C) as well as the whole genome (Figure S1, red shading). After selection, the separation in distances between like and unlike types becomes very distinct (Figure 2B, D), even for closely related cell types (Figure 3). These regions similarly highlight distinctions between cell types both in correlations (Figure S3, S4, S5, S6, S7, S8), and distance matrices between cell types (Figure S9, S10, S11, S12).

Since Monte Carlo selection is stochastic, different sets of fingerprinting regions can be selected in different runs. To evaluate the stability of regions included in replication fingerprints, we applied the algorithm 100 times for each type of human and mouse fingerprint constructed (Figure S13). Results demonstrate that fingerprinting regions are well-conserved among multiple rounds of selection, with the top 10–14 regions selected in 100/100 trials in each case. For all subsequent classification, we used regions included in at least 75/100 fingerprinting runs.

As the distances between profiles derive from either the same or different cell types (Figure 2C), their distributions can be used to create a general classifier (Figure 2C,D, Figure 3A), with an error rate proportional to the overlap in distances shared by “like” and “unlike” cell type comparisons (Figure 2C,D, blue shading). This allows us to state a level of confidence for a given prediction, as well as estimate the similarity of a cell type to others. To refine this classification, we applied the k-nearest-neighbor rule [25] (kNN; k = 3) to assign cell types according to the three most similar profiles in the training set. Distances below the threshold – θ = 2.4 in Figure 2D – are hypothesized to derive from similar cell types, and are used with kNN to classify profiles according to the closest profiles in the training set. Distances above the threshold are presumed to derive from different cell types, preventing kNN from classifying highly divergent RT profiles as the cell type of the most similar known profile.

### Classification of cell types using fingerprint regions

To test the ability of our method to select suitable regions for classification, we applied it to predict the known identity of 9 mouse and 7 human cell types with 36 and 31 total experimental replicates, respectively. Datasets used for prediction are summarized in Table S1, with most described in detail in previous publications [3]–[5]. Rough classification of each experiment into like and unlike cell types by a distance ratio cutoff was accurate in 951/961 (99.0%) human and 1250/1296 (96.5%) mouse comparisons respectively (Figure 3A,B). Refining this classifier by using kNN to assign cell types according to the three most similar profiles in the training set resulted in correct predictions for 36/36 mouse and 31/31 human replication timing profiles (Figure 3C,D). Strikingly, even closely related cell types could be reliably distinguished using this method, such as mouse ESCs and early primitive ectoderm-like stem cells (EPL/EBM3), and two day intermediates of human ESC differentiation into endomesoderm (DE2; day 2) and definitive endoderm (DE4; day 4). Thus, replication profiles appear capable of distinguishing among a wide array of cell types in early mouse and human development.

### Confirmation and generalizability of replication fingerprints

The use of all experimental data in a selection algorithm often results in overfitting the model to a limited set of observations. For this reason, machine-learning algorithms are commonly trained and tested on different subsets of data (termed cross-validation). To determine whether overfitting is occurring in our selection method and assess the degree to which fingerprinting domains are generally cell type-specific, we performed leave-one-out cross-validation (LOOCV) with each of the available experiments by constructing fingerprints using all but one experimental replicate, and testing classification on the remaining replicate. In all cases (31/31 human, 36/36 mouse), correct predictions in the excluded profile confirmed that fingerprinting regions remained consistent with cell type, and that most cell-line-specific differences were discarded (Figure 3C, LOOCV column). This was also true for a cell line with only one replicate (mouse 46C neural precursor cells), implying that most of the regions of differential replication timing useful for classification are shared between cell lines.

To simulate the classification of a cell type not yet encountered in the training set, we tested predictions after selecting fingerprinting regions with all replicates of a given cell type excluded (Figure 3C, LCTO column). This confirmed that most cell types not yet encountered were correctly classified as “Unseen” (7/7 cell types in human, 7/9 in mouse). However, two cases in which profiles were ambiguous were between neural precursors (NPCs) and mouse epiblast-like stem cells (EpiSCs, EBM6), suggesting that closely related cell types are more accurately distinguished when examples of each type are included in the training set.

### A replication fingerprint for pluripotency

**Figure 4:**
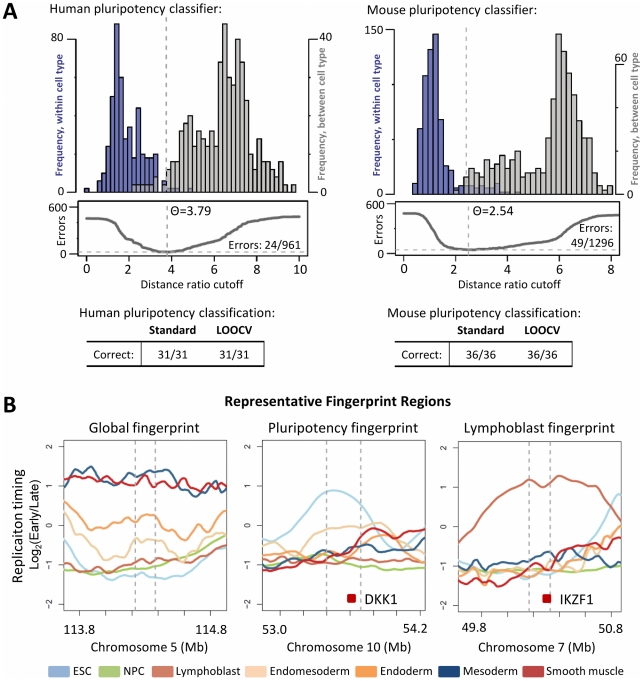
Identification of cell type- and pluripotency-specific regions. **A.** Construction of a general classifier for distinguishing pluripotent from committed mouse and human cell types, with results summarized in the tables below for the standard kNN method and leave-one-out crossvalidation. **B.** Representative fingerprint regions are shown for three cases: general classification (left), distinguishing pluiripotent vs. committed cell types (middle), and identifying cell-type-specific (here, lymphoblast-specific) regions (right). Lines represent averaged profiles for each cell type. Several EtoL regions in the pluripotency fingerprint contain genes known to function in maintaining stem cell identity, such as Dickkopf homolog DKK1, while uniquely early regions in cell type-specific fingerprints often feature genes with relevant functional or disease associations, such as IKZF1 in lymphoblast cells.

One of the most striking features of replication timing is its widespread consolidation into larger replication domains during neural differentiation, concomitant with global compaction of chromatin [3], [4]. This consolidation, along with recovery of ESC replication timing by induced pluripotent stem cells (iPSCs), suggested that replication patterns in specific regions of the genome are associated with the pluripotent state. Further, if certain timing changes are a stable property of cellular commitment, they may provide a unique opportunity to evaluate differentiation capacity using replication-timing patterns. To explore this, we analyzed the differences in replication profiles between collections of pluripotent/reversible (ESCs, iPSCs, EBM3) and committed cell types in 13 human and 21 mouse cell lines (Figure 4A). In each case, we created a stringent consensus fingerprint for classification consisting of regions found in >75/100 runs (18 regions each in mouse and human), and examined genes in the top 200 fingerprint regions (∼2% of the genome) to characterize a more inclusive sample. Genes and regions found to consistently switch to earlier or later replication as pluripotency is lost are provided in Tables S3, S4, S5, S6.

Strikingly, several regions displayed conserved, significant differences in timing between all pluripotent and committed cell types (Figures 4A, S10, S12). As with general fingerprints, classification into pluripotent or committed types could be performed unambiguously (36/36 cases in mouse, 31/31 in human), even with regions selected with the test profile excluded (LOOCV column). Several of the genes consistently switching to later replication in mouse and human pluripotency fingerprints have known roles in maintaining pluripotency (for instance, Dppa2 and Dppa4 in both species, and DKK1 in human; Tables S4 and S6). In addition, two classes of genes stood out from this analysis that showed significant switches to later replication in both species: a large cluster of protocadherins (PCDs), and the majority of the Hist1 cluster of core histone genes (Table S7). The former are developmentally regulated genes with broad involvement in neural development and cell-cell signaling [26], [27], and switch to later replication in all committed mouse and human cell types. The latter Hist1 cluster was later replicating in 8/8 committed cell types in mouse and 5/6 in human (not lymphoblasts), and includes several core histone genes that were downregulated up to 2.5-fold in NPCs. These results are intriguing in light of previous reports of histone downregulation during development [28], as well as a hyperdynamic chromatin phenotype in ESCs that involves higher exchange rates of histone H1 [29] and is required for efficient somatic cell nuclear reprogramming in *Xenopus* oocytes [30]. Importantly, all of the histone H1 genes are found in this cluster, suggesting that regulation of global H1 abundance may provide a mechanism for the overall chromatin compaction and consolidation of replication timing observed during neural differentiation [3]–[5].

**Figure 5:**
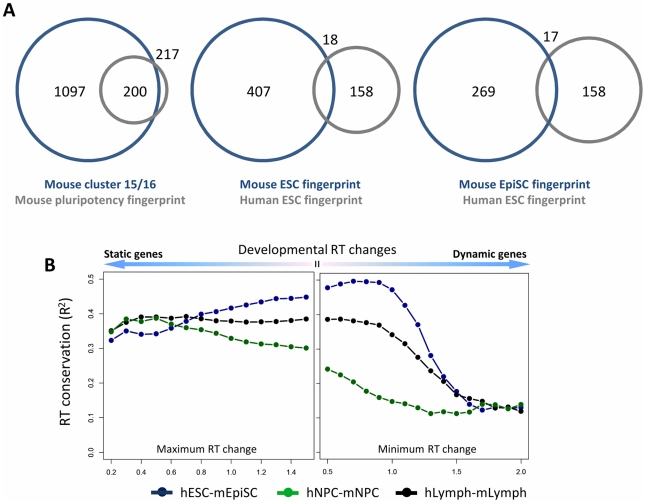
Conservation of mouse and human pluripotency fingerprint genes. **A.** Venn diagram showing the overlap in genes that fail to reprogram expression in partial iPSCs (clusters 15 and 16 in Hiratani *et al.*, 2010) and the mouse pluripotency fingerprint (left), between the human and mouse ESC fingerprints (middle), and the human ESC and mouse EpiSC fingerprint (right). **B.** Conservation (R^2^) of replication timing between human and mouse lymphoblasts (hLymph-mLymph), neural precursors (hNPC-mNPC) and primed stem cells (hESC-mEpiSC) as a function of developmental timing changes. For the most closely aligned samples, both relatively static and highly dynamic regions show a decreased alignment in replication timing between species.

To characterize the genes included in the mouse pluripotency fingerprint, we compared them to a previous class of genes that showed lineage-independent switches to later replication in mouse ESC differentiation, and failed to revert to ESC-like expression in three separately derived samples of partial iPSCs (clusters 15 and 16 in Figure 7 of Hiratani et al., 2010). Remarkably, 200 out of 217 genes in the top 100 mouse pluripotency regions belonged to this class, despite very different methods for deriving them (Figure 5A). All of the fingerprint genes switched to later replication, and at the transition between early and late epiblast stages where cell fates become restricted [5]. Most genes also had reduced expression in late epiblast and neural progenitor stages (average 1.66-fold reduction in transcription from ESC/EBM3 to EBM6/NPCs). Thus, some of these genes may make prime candidates for improving the efficiency of iPSC production, or for reverting human ESCs to a more naive, mouse ESC-like state. However, the overlap between human and mouse pluripotency fingerprint genes, while significant, was much lower (Figure 5A), and this was true even when comparing human ESCs to developmentally analogous mouse EpiSCs [3], [31]. Therefore, many pluripotency-associated genes and loci may be species-specific, consistent with recent studies that underscore considerable differences between mouse and human pluripotency networks [32], [33]. This low alignment is also accounted for by a general drop in overall alignment in regions with the greatest developmental switches in replication timing (Figure 5B), which are those preferentially selected by the fingerprinting algorithm.

Of the genes conserved in the fingerprints of both species (indicated by boldface type in Tables S4 and S6), most belong to the aforementioned large class of protocadherins. However, Dppa2 and Dppa4 are also conserved, as well as genes with no known roles in maintaining pluripotency (Cast, Riok2, Lix1) that reside within the same replication units as pluripotency fingerprint genes in both species. Other core pluripotency genes remain relatively early replicating in both species (Pou5f1[Oct4], Sox2, Nanog), and are likely regulated by other mechanisms. For instance, Sox2 belongs to a class of genes with strong promoters (HCP, or high CPG content promoters) generally unaffected by local replication timing [4], [34].

### Independent verification of fingerprint classification by PCR

**Figure 6:**
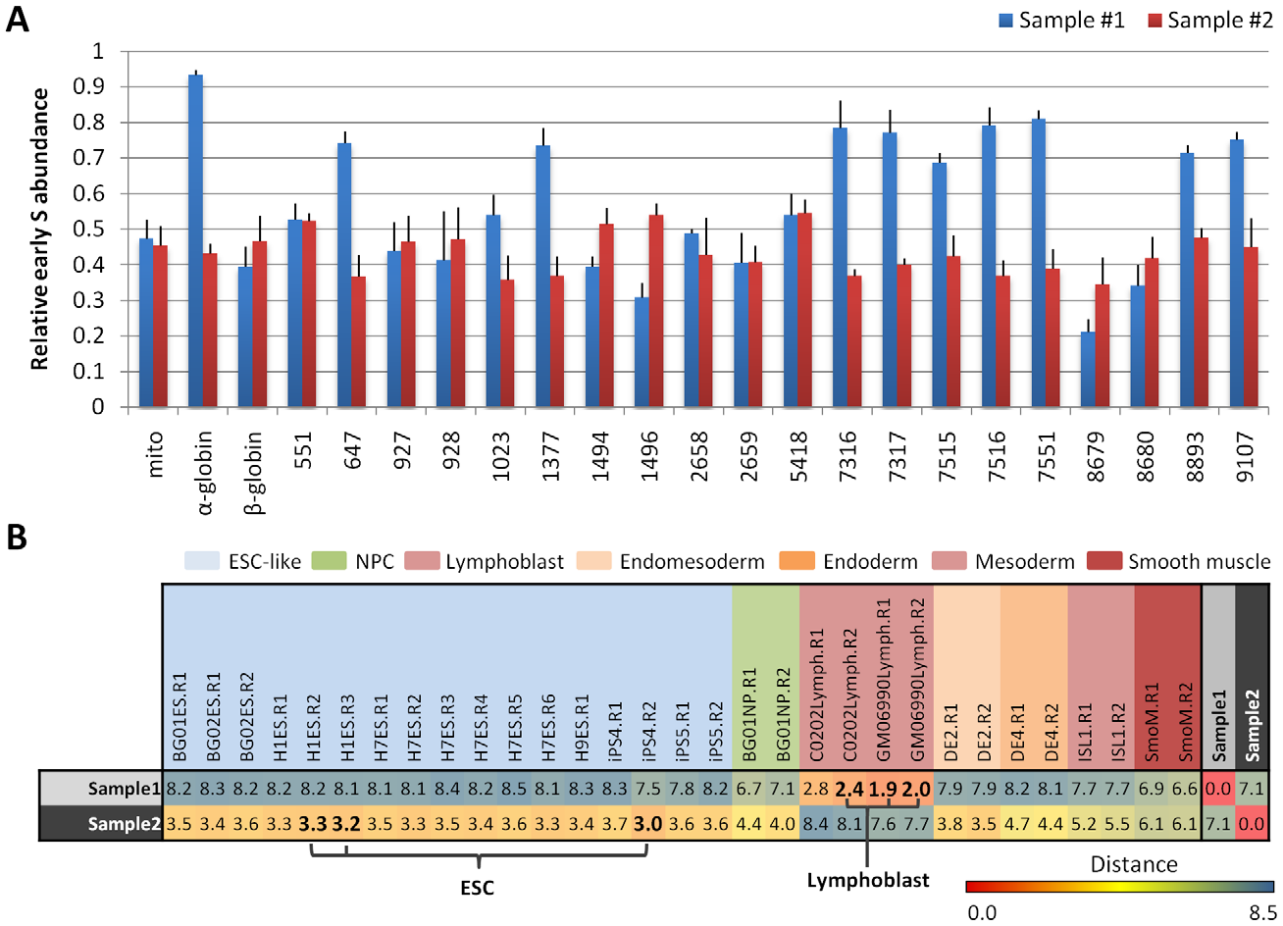
Independent verification of fingerprint classification by PCR. **A.** NC-NC lymphoblasts and WIBR3 hESCs were BrdU labeled, early and late nascent strands were purified as for all other cells, and nascent strands were analyzed blindly by PCR using primers specific to 20 human fingerprint regions and control regions (mito: mitochondrial DNA, α-globin, β-globin). Replication times are represented by the relative abundance of each sequence in early S phase as a fraction of its abundance in both early and late S. Error bars depict the average and SEM for each locus after 6 replicate experiments. **B.** Euclidean distances between replication profiles measured in fingerprint regions described in Table 1, after rescaling PCR values to array scale. Color scale for numbers relates the relative similarity of cell types in fingerprint regions, from highly similar (red) to highly divergent (blue). The three lowest distances used for kNN classification (k = 3) are highlighted in bold font, with unknown samples #1 and #2 correctly designated as lymphoblasts and ESCs, respectively using the three shortest distances.

One potential application of replication fingerprints is in the development of PCR kits for epigenetic classification, particularly for cell types or disease samples with no known aberrations in transcription or sequence. To confirm that fingerprint regions can be translated into a classification scheme using site-specific PCR, we classified two unknown samples representing cell types that were analyzed previously, but that were derived from different cell lines than the original set used for training. The experiment was performed in a blind manner in which the experimenter had no prior knowledge of the regions or cell types being tested. Primers were assembled against sequences within 10–20 kb from the center of each fingerprint region, and the replication times of each region were quantified as the “relative early S phase abundance” (relative abundance of a sequence in nascent strands from early S phase), as previously described [35] (Figure 6A). PCR-based timing values were rescaled for consistency with the original scale of the array datasets used in training, and distances were calculated between the unknown samples and other human profiles in fingerprint regions (Figure 6B). Using the same methods as in prior classifications, these distances correctly identified the two unknown samples as lymphoblasts and hESCs, respectively; the three known datasets with the smallest distances were each of the correct cell type.

## Discussion

### Advantages and caveats of replication profiles for cell typing

Our method for cell typing through replication fingerprinting addresses a well-recognized need for comprehensive methods to assess cellular identity and differentiation potential in stem cell biology. Unlike other molecular markers, replication is regulated at the level of large, multi-megabase domains, making comprehensive, genome-wide profiles relatively simple to generate and interpret [36]. In particular, the robust stability of replication timing profiles in stem cells [8], and wide divergence between cell types make them a promising candidate for classification.

While the functional role for the replication program is not yet understood, its conservation between human and mouse cell culture models of development support its functional significance. We and others have shown a substantial correlation (R^2^ = 0.42–0.53) in replication patterns between mouse and human cell types, with timing patterns of embryonic stem cells, neural precursor cells, and lymphoblastoid cells most closely aligned to their cognate in the other species [1], [3]. The important role for replication is further corroborated by its remarkably strong link to genome organization [3], and its ability to confirm the mouse epiblast identity of human ESCs genome-wide and with an epigenetic property [3], [31].

By comparison, methods for cell typing using DNA methylation, gene expression, histone modifications or protein markers are well suited to some applications [10]–[16], but may not be informative for certain fractions of the genome, or may rely on genome features that cannot distinguish between similar cell states. We therefore envision replication fingerprinting as a complement to existing cell typing strategies that may be used for samples unsuitable for traditional methods, or for additional confidence in assessing cell identity in cases where this is critical, such as regenerative medicine. One caveat to consider in these applications is that replication profiles, similar to other genome-wide methods, are an ensemble aggregate from many cells, making measurement of homogeneity difficult. In addition, as with other supervised classification approaches, the method is informative only for cell types (classes) available during training. However, our fingerprinting method is in principle applicable to any data type, and may be modified to select discriminating features in other epigenetic profiles.

A major advantage of our fingerprinting method is in selection of a minimal set of regions that allow for classification with a straightforward PCR-based timing assay and a reasonably small set of primers, particularly if only cell-type specific regions are examined. Our results suggest that a standard set of 20 fingerprint loci can be effective for classification, but the number of regions queried can be adjusted based on the confidence level required. The sole requirement for replication profiling is the collection of a sufficient number of proliferating cells for sorting on a flow cytometer. Consistently, just as replication fingerprints can be generated for particular cell types or general categories of cells, features of replication profiles allow for the creation of disease-specific fingerprints, which may be valuable for prognosis.

### Consistent timing changes between pluripotent and committed cell types

In addition to cell typing applications, replication profiling is informative for basic biological questions. Here, we have identified regions that may undergo important organizational changes upon differentiation, which include a class of gene that fail to reverse expression in partial iPSCs, and the majority of mouse and human histone H1 genes. Human lymphoblasts retained early replication in H1 genes, which may be explained by their high rate of proliferation. Since highly developmentally plastic regions (including pluripotency fingerprint regions) are poorly conserved (Figure 5B) the evolutionary conservation of cell-type specific timing patterns must be driven by the moderately changing majority of the genome.

The recent derivation of mouse ESC-like human stem cells with various methods raises an intriguing question [37]: will naïve hESCs align better to mESCs than to mEpiSCs for replication timing as they have for transcription? Although pluripotency is currently assessed by marker gene expression or laborious complementation experiments, replication timing assays in regions uniquely early or late replicating in pluripotent cells provide a tractable method to predict the pluripotency of various cell types, as well as insights into conserved genome organizational changes during differentiation.

## Methods

### Cell culture and differentiation

Mouse replication timing datasets are described in Hiratani et al., 2010. Briefly, mouse embryonic stem cells (ESCs) from D3, TT2, and 46C cell lines were subjected to either 6-day (46C) or 9-day (D3, TT2) neural differentiation protocols to generate neural progenitor cells (NPCs) [4], [5]. For D3, intermediates were also profiled after 3 (EBM3) and 6 (EBM6) days of differentiation. Muscle stem cells (myoblast) and induced pluripotent stem cells (iPSCs) reprogrammed from fibroblasts were collected as described for human and mouse [38]–[40]. For human timing datasets, neural precursors were differentiated from BG01 ESCs as described in Schulz et al., 2004 [3], [41]. Lymphoblast cell lines GM06990 and C0202 were cultured as previously described [2], [42]. Differentiation of BG02 hESCs to mesendoderm (DE2) and definitive endoderm (DE4) was performed by switching from defined media (McLean et al. [20]) to DMEM/F12+100 ng/ml Activin A 20 ng/ml Fgf2 for two and four days, respectively, with 25 ng/ml Wnt3a added on the first day. Mesoderm and smooth muscle cells were derived by adding BMP4 to DE2 cells at 100 ng/ml.

### Generation and preprocessing of microarray datasets

Using custom R/Bioconductor scripts [43], [44], microarray data from Hiratani et al. 2008, Hiratani et al. 2010, and Ryba et al., 2010 were normalized to equivalent scales, and averaged in nonoverlapping windows of approximately 200 kb. Additional profiles for human ESCs, definitive endoderm, mesendoderm, mesoderm, and smooth muscle were derived, normalized and scaled equivalently, as described [45]. Profiles shown in Figure 1 and Figure 4 were smoothed using LOESS with a span of 300 kb.

### Monte Carlo selection of fingerprinting regions

Selection of fingerprint regions was performed as described using custom R/Bioconductor scripts. Regions of non-conserved RT (2000/10994 mouse, 2000/12625 human) were first selected based on standard deviation, then optimized using a Monte Carlo algorithm (Figure S2). Using the Metropolis-Hastings criterion for Monte Carlo with simulated annealing [23], [24], moves are accepted when exp((dR_best_−dR)/T)>i, where dR is the distance ratio of the proposed move, dR_best_ is the current best distance ratio, T is a temperature parameter that decreases geometrically during the simulation, and i is a random number from 0 to 1.

### Cell type classification

Cell type classification was performed using absolute distances between experiments measured from replication timing in fingerprint regions, using the k-nearest neighbor rule with k = 3; *i.e.*, each profile was categorized according to the three nearest profiles. Crossvalidation was performed to select an appropriate value for k, with k = 3 chosen as the smallest value that yielded 100% classification accuracy after leave-one-out crossvalidation (LOOCV) to allow classification of cell types with fewer replicates. For LOOCV results, each experiment was sequentially left out during Monte Carlo selection, and the resulting regions were used to predict the identity of the excluded experiment. To test prediction on cell types not yet encountered, all profiles for a given cell type were left out during region selection (LCTO), and cell type was predicted using the resulting regions. All data analysis was performed using custom R scripts and Bioconductor packages [43], [44].

### Cell type classification using PCR

For each fingerprint region depicted in Table 1, 10–20 kb from the center of the region was sent to NCBI Primer-Blast (http://www.ncbi.nlm.nih.gov/tools/primer-blast/) to design several PCR primer sets with product sizes of 150–350 bp, using standard parameters. Forward and reverse primer pairs displaying the greatest specificity were chosen. Primer sets were verified for specificity and product size using the In-Silico PCR tool at the UCSC genome browser (http://genome.ucsc.edu/cgi-bin/hgPcr).

**Table 1:** Primers used for PCR fingerprint verification.

					Genomic region		
Region	F/R	Sequence	Length	Product region (Hg18)/Size	Chr	Start	End
551	Forward	ACATGGGCGTGCAATCCCCA	20	chr1:145,442,862–145,443,081	chr1	145,439,397	145,449,397
	Reverse	GGCCCTGGTTGCTAGGTGCG	20	220			
647	Forward	GCAAAACGGCCAACGGCTGA	20	chr1:167,999,361–167,999,518	chr1	167,993,179	168,003,179
	Reverse	GGCCTGCCAGTGCTGAGAGG	20	158			
927	Forward	TTGGCCCACGTGTGGGGAGA	20	chr1:230,199,814–230,200,011	chr1	230,199,352	230,209,352
	Reverse	TGACCCCCTCCCAGGCAGTG	20	198			
928	Forward	TGACCCCTGCACCCACCACA	20	chr1:230,387,991–230,388,261	chr1	230,396,329	230,406,329
	Reverse	GCCGCAGCTGGAAAAGGGGT	20	271			
1023	Forward	GCGTTGCCCTTTGCCACCAC	20	chr10:4,017,971–4,018,253	chr10	4,008,411	4,018,411
	Reverse	GCTGCAGCCTCCACCTGCAA	20	283			
1377	Forward	AGCCCTGAAAGGTGAGCCCCA	21	chr10:90,168,738–90,168,918	chr10	90,159,026	90,169,026
	Reverse	GGGTGGGAGGGGGCTGCTAA	20	181			
1494	Forward	GCACGTTGCAGATGCACTGCG	21	chr10:114,445,426–114,445,576	chr10	114,440,981	114,450,981
	Reverse	TGGATGGGTCACGTGTGGCG	20	151			
1496	Forward	GTTGCTCACCACGGGAGGCC	20	chr10:114,788,086–114,788,296	chr10	114,782,400	114,792,400
	Reverse	CCACAAGCCAGCCGACGGAG	20	211			
2658	Forward	GGGTTTCCGGGCAGGTGGTG	20	chr12:114,297,381–114,297,554	chr12	114,296,629	114,306,629
	Reverse	AGCCCCGGCTTTCCTCCCTT	20	174			
2659	Forward	CCGCCTCTTCCCACCCCACT	20	chr12:114,463,910–114,464,159	chr12	114,463,748	114,473,748
	Reverse	GGCCTGGCAGGGGTTTTGCT	20	250			
5418	Forward	GTGGGGATTGACTGCACTGCCA	22	chr2:44,479,986–44,480,137	chr2	44,479,922	44,489,922
	Reverse	TCAATGCCCCCTCCCCCAACA	21	152			
7316	Forward	GTGCCCAGCGGCCAGTGTAG	20	chr3:109,192,760–109,192,989	chr3	109,187,624	109,197,624
	Reverse	CTGAGTGTGGCGCCTGTGGG	20	230			
7317	Forward	CGCCCTATCCCGGGACTGCT	20	chr3:109,390,861–109,391,033	chr3	109,380,748	109,390,748
	Reverse	CCTGTGTCTGCTTCCCCCACC	21	173			
7515	Forward	AAGGCCAGTTGCAGGCCCTG	20	chr3:153,125,829–153,126,087	chr3	153,111,466	153,121,466
	Reverse	CACCCCAACGGCGCATGGTT	20	259			
7516	Reverse	AGGCAGCACTGCGGTAATATGCT	23	chr3:153,356,058–153,356,339	chr3	153,355,644	153,365,644
	Forward	GGGCTGCAGTGGGTTCTGCC	20	282			
7551	Reverse	GTGGCAGCTACACGGCCAGG	20	chr3:161,085,915–161,086,106	chr3	161,084,943	161,094,943
	Forward	GGAAGCCCCAAACCCAGGCA	20	192			
8679	Reverse	TCTGTGCGGGCCTGAAGGGG	20	chr5:38,608,473–38,608,822	chr5	38,601,124	38,611,124
	Forward	AGCGGCCACATCTTGCAGGT	20	350			
8680	Reverse	GGCACCACGAGGGAGATGCG	20	chr5:38,794,843–38,795,022	chr5	38,786,446	38,796,446
	Reverse	CCCGGCAGTGGGAGAGACGA	20	180			
8893	Forward	CTGGCCCCTCCTCACCTCCG	20	chr5:95,884,467–95,884,676	chr5	95,902,422	95,912,422
	Reverse	GGCACACCAGGCAGCACCTC	20	210			
9107	Forward	ACACATTTGCTGAGGGCCCACT	22	chr5:142,903,434–142,903,692	chr5	142,899,586	142,909,586
	Reverse	ACTGTCCCATCCTGCGGCCT	20	259			

PCR reactions were set up using 1.25 ng genomic DNA and 1 uM each of forward and reverse primers in 12.5 uL scaled according to the instructions of Crimson Taq DNA Polymerase (NEB). Thirty six cycles of PCR (empirically determined to be unsaturated for amplification) were performed according to manufacturer's conditions with annealing temperature of 62°C. One-third of the reaction was analyzed on a 1.5% agarose gel containing ethidium bromide. The gel was scanned by Typhoon Trio (GE Healthcare) and band intensity was quantified by Image Quant TL (GE Healthcare). After the background was subtracted, signal intensity from the early S fraction was divided by the sum of those from early S and late S fractions from each sample, as described [35]. PCR timing values were converted to array RT scale (equivalent root-mean-square) using the scale function in R, and distances were calculated against other cell types as previously performed.

### Accession numbers

GSE18019, GSE20027

## Supporting Information

Figure S1 **Classification errors using whole genome nearest neighbor approach.** The above distances were calculated between profiles as in Figure 2, using the entire genome rather than an optimized set of fingerprinting regions. Classification errors (shaded red) result when distances between cell types are smaller than the distance within cell types. Here, TT2 ESC replicate 1 could be misclassified as an NPC, or D3 NPC replicate 2 as an ESC.
(TIF)

Figure S2 **The Monte Carlo optimization algorithm.** A. Regions used in replication fingerprints are selected using a two step algorithm. First, 200 kb segments with significant changes in replication timing between any two cell types are isolated. Next, a random set of these segments are sampled to calculate a distance ratio (Figure 1C) representing the starting separation between cell types, and an iterative algorithm randomly selects between one of three moves: 1) include an unused region in the fingerprint, 2) remove a region from the fingerprint, or 3) swap regions between fingerprint and unused lists. By the Metropolis-Hastings criterion, moves that improve the separation between cell types (increase the distance ratio criterion) are accepted with a higher probability than those that do not. B. Maximization of the distance ratio (left) as domain number (right) decreases to a predetermined minimum (here, n = 20).
(TIF)

Figure S3 **Genomewide correlations between mouse timing datasets.** Heatmaps depict the level of correlation between timing datasets averaged in 200 kb windows, from low (red) to high (white). Note the relatively high level of variation in correlations between similar and divergent cell types (compare to Figure S4).
(TIF)

Figure S4 **Correlations between mouse timing datasets in consensus cell-type fingerprint regions.** Heatmaps depict the level of correlation between timing datasets in 200 kb fingerprint regions. from low (red) to high (white). Compare with Figure S3.
(TIF)

Figure S5 **Correlations between mouse timing datasets in consensus pluripotency fingerprint regions.** Heatmaps depict the level of correlation between timing datasets in 200 kb fingerprint regions, from low (red) to high (white).
(TIF)

Figure S6 **Genomewide correlations between human timing datasets.** Heatmaps depict the level of correlation between timing datasets averaged in 200 kb windows, from low (red) to high (white). Note the relatively high level of variation in correlations between similar and divergent cell types (compare to Figure S7).
(TIF)

Figure S7 **Correlations between human timing datasets in consensus cell-type fingerprint regions.** Heatmaps depict the level of correlation between timing datasets in 200 kb fingerprint regions, from low (red) to high (white).
(TIF)

Figure S8 **Correlations between human timing datasets in consensus pluripotency fingerprint regions.** Heatmaps depict the level of correlation between timing datasets in 200 kb fingerprint regions, from low (red) to high (white).
(TIF)

Figure S9 **Distance matrix for mouse cell type consensus fingerprint.** Numbers indicate the Euclidean distance between replication profiles measured in the 18 regions included in over 75% of runs of the fingerprinting algorithm. Cell type definitions used for training are indicated by the color map in rows and columns (see color key at top). Color scale for distances relates the relative similarity of cell types in fingerprint regions, from highly similar (red) to highly divergent (blue).
(TIF)

Figure S10 **Distance matrix for mouse pluripotency consensus fingerprint.** Numbers indicate the Euclidean distance between replication profiles measured in the 18 regions included in over 75% of runs of the fingerprinting algorithm. Cell type definitions used for training are indicated by the color map in rows and columns (light blue: pluripotent cell types; dark blue: committed cell types). Color scale for numbers relates the relative similarity of cell types in fingerprint regions, from highly similar (red) to highly divergent (blue).
(TIF)

Figure S11 **Distance matrix for human cell-type consensus fingerprint.** Numbers indicate the Euclidean distance between replication profiles measured in the 18 regions included in over 75% of runs of the fingerprinting algorithm. Cell type definitions used for training are indicated by the color map in rows and columns (see color key at top). Color scale for numbers relates the relative similarity of cell types in fingerprint regions, from highly similar (red) to highly divergent (blue).
(TIF)

Figure S12 **Distance matrix for human pluripotency consensus fingerprint.** Numbers indicate the Euclidean distance between replication profiles measured in the 18 regions included in over 75% of runs of the fingerprinting algorithm. Cell type definitions used for training are indicated by the color map in rows and columns (light blue: pluripotent cell types; dark blue: committed cell types). Color scale for numbers relates the relative similarity of cell types in fingerprint regions, from highly similar (red) to highly divergent (blue).
(TIF)

Figure S13 **Calculation of consensus fingerprint regions.** Since the Monte Carlo algorithm will randomly include or exclude regions in each run, the suitability of a set of regions for classification can be evaluated by running the algorithm multiple times and choosing the regions most often present. Regions with particularly unique timing in each cell type are often selected in 100/100 trials; here, we select regions included in at least 75 out of 100 runs for ‘consensus’ fingerprints for mouse and human cell type and pluripotency regions. The x-axis depicts the rank of each region in percentage of runs with that region included.
(TIF)

Table S1 **Summary of experimental datasets.** References and brief descriptions for each cell line and cell type analyzed.
(XLSX)

Table S2 **Window size comparison.** A summary of algorithm performance using window sizes of 50 kb, 100 kb, 200 kb, and 400 kb. Windows of 200 kb were used for the remaining analyses to correspond with the unit size of developmental replication timing changes, which is typically 400–800 kb [3]–[5].
(XLSX)

Table S3 **Mouse pluripotency fingerprint regions.** Genomic locations (from the mm8 build) and average replication timing values for the top 200, 100, and 20 pluripotency fingerprint regions for mouse cell types.
(XLSX)

Table S4 **Mouse pluripotency fingerprint genes.** Replication timing and transcription values for genes included in the top 200, 100, and 20 mouse pluripotency fingerprint regions. Genes in common with the human pluripotency fingerprint are highlighted in bold font. Timing values are assigned to transcription start loci from loess smoothed profiles with a span of 300 kb.
(XLSX)

Table S5 **Human pluripotency fingerprint regions.** Genomic locations (from build hg18) and average replication timing values for the top 200, 100, and 20 pluripotency fingerprint regions for human cell types.
(XLSX)

Table S6 **Human pluripotency fingerprint genes.** Replication timing and transcription values for genes included in the top 200, 100, and 20 human pluripotency fingerprint regions, with genes in common with the mouse pluripotency fingerprint highlighted in bold font.
(XLSX)

Table S7 **Human and mouse HIST1 genes.** Replication timing and transcription values across genes in the major HIST1 cluster in human and mouse.
(XLSX)
