# Supplementary figures and images for: Replication Timing: A Fingerprint for Cell Identity and Pluripotency
Source: PLoS Comput Biol. 2011 Oct 20;7(10):e1002225. doi: 10.1371/journal.pcbi.1002225 (PMC3197641; doi:10.1371/journal.pcbi.1002225)

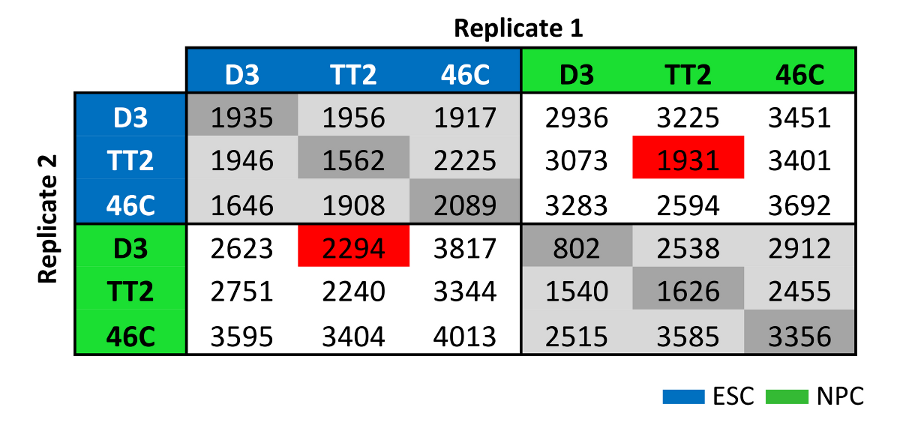

Supplement: Figure S1 — Classification errors using whole genome nearest neighbor approach. The above distances were calculated between profiles as in Figure 2, using the entire genome rather than an optimized set of fingerprinting regions. Classification errors (shaded red) result when distances between cell types are smaller than the distance within cell types. Here, TT2 ESC replicate 1 could be misclassified as an NPC, or D3 NPC replicate 2 as an ESC. (TIF) [file pcbi.1002225.s001.tif]

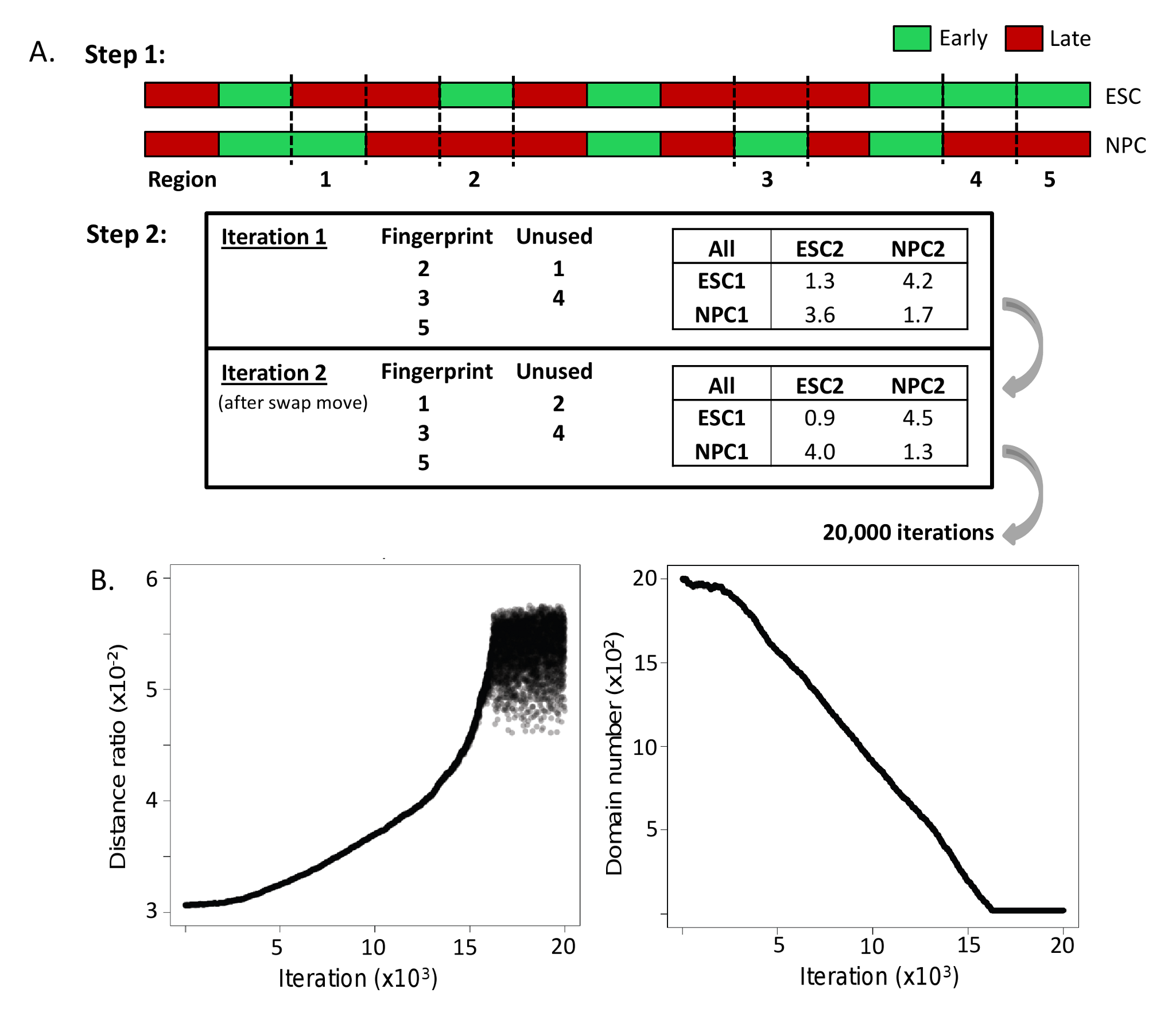

Supplement: Figure S2 — The Monte Carlo optimization algorithm. A. Regions used in replication fingerprints are selected using a two step algorithm. First, 200 kb segments with significant changes in replication timing between any two cell types are isolated. Next, a random set of these segments are sampled to calculate a distance ratio (Figure 1C) representing the starting separation between cell types, and an iterative algorithm randomly selects between one of three moves: 1) include an unused region in the fingerprint, 2) remove a region from the fingerprint, or 3) swap regions between fingerprint and unused lists. By the Metropolis-Hastings criterion, moves that improve the separation between cell types (increase the distance ratio criterion) are accepted with a higher probability than those that do not. B. Maximization of the distance ratio (left) as domain number (right) decreases to a predetermined minimum (here, n = 20). (TIF) [file pcbi.1002225.s002.tif]

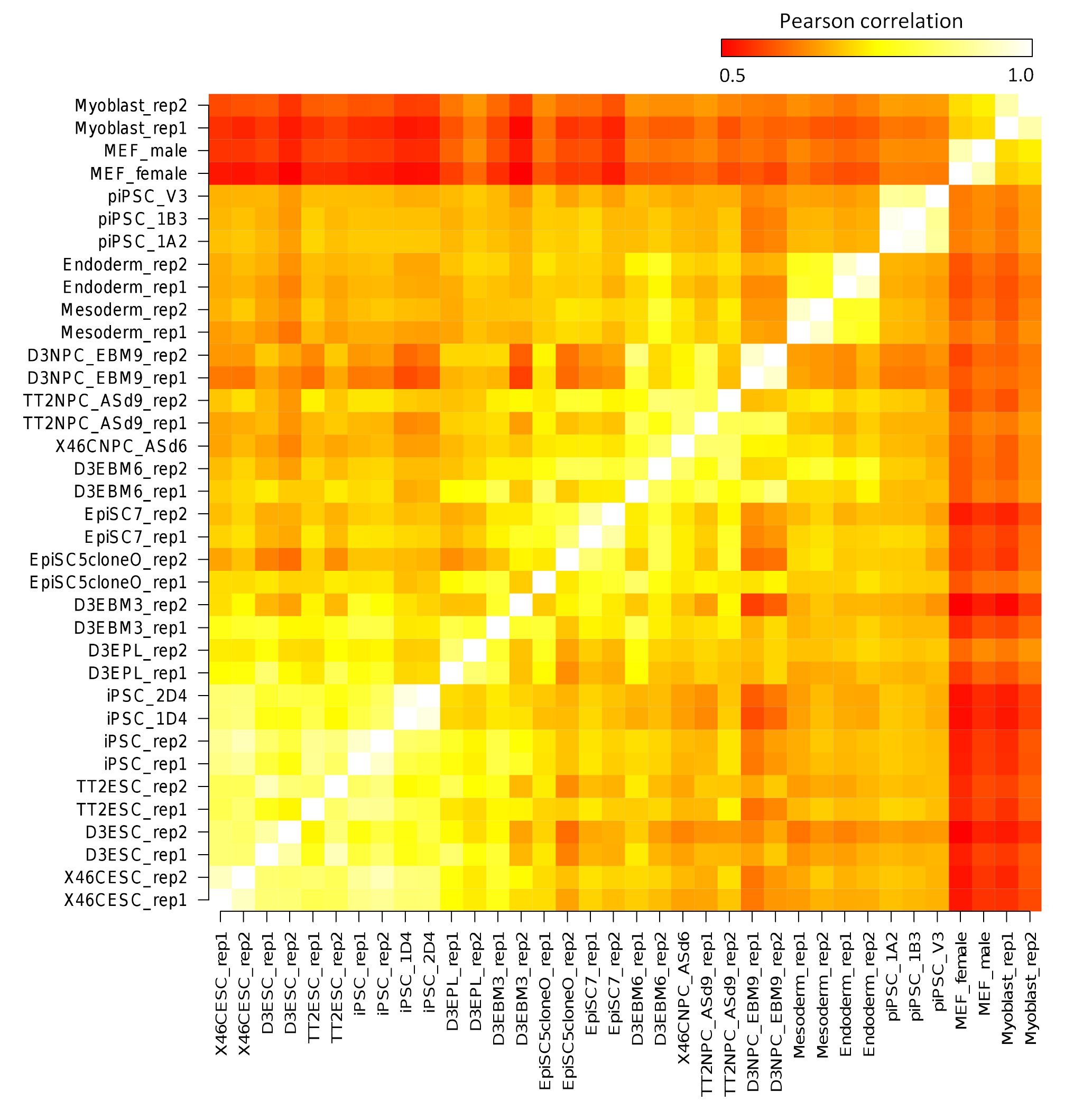

Supplement: Figure S3 — Genomewide correlations between mouse timing datasets. Heatmaps depict the level of correlation between timing datasets averaged in 200 kb windows, from low (red) to high (white). Note the relatively high level of variation in correlations between similar and divergent cell types (compare to Figure S4). (TIF) [file pcbi.1002225.s003.tif]

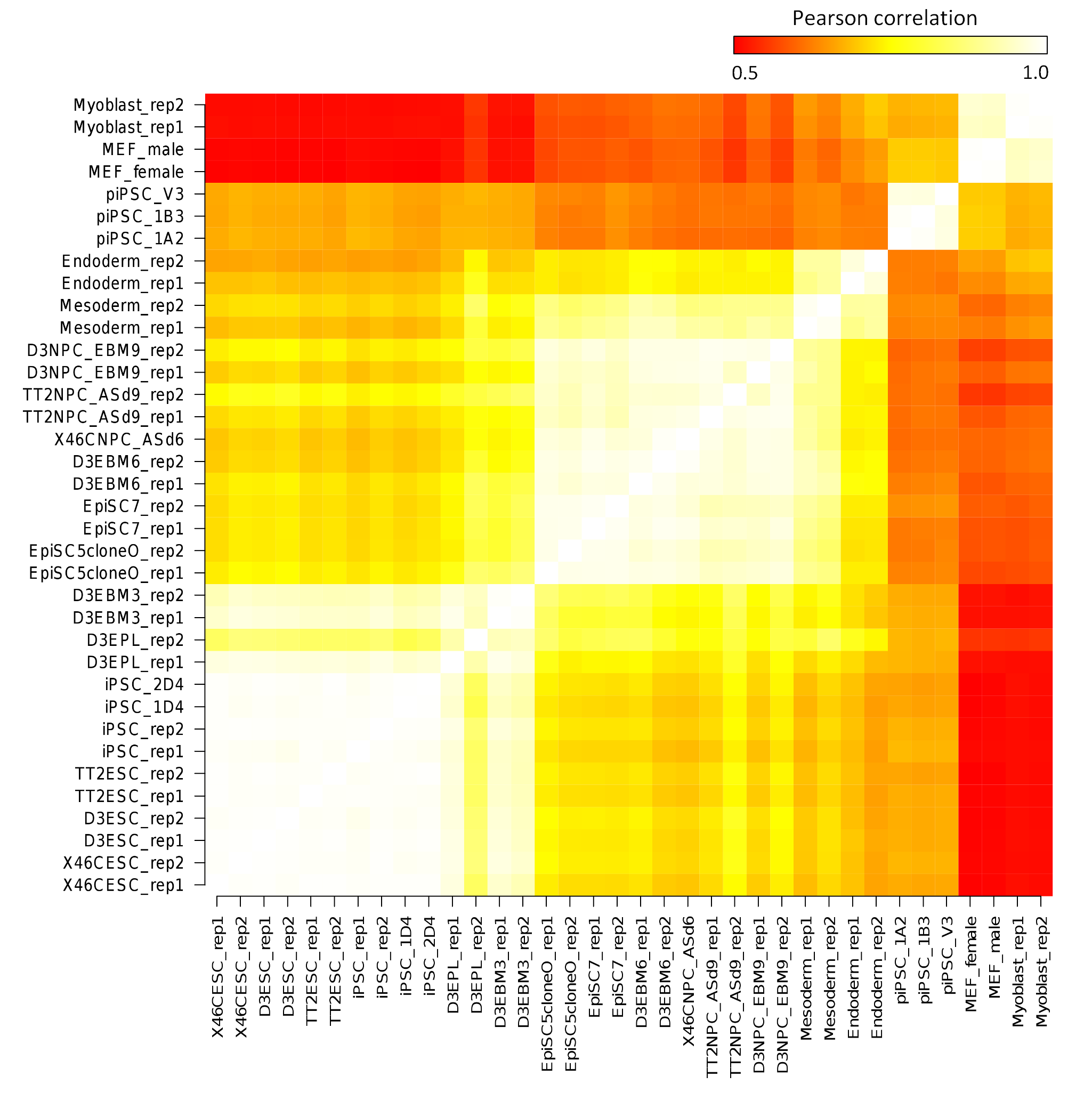

Supplement: Figure S4 — Correlations between mouse timing datasets in consensus cell-type fingerprint regions. Heatmaps depict the level of correlation between timing datasets in 200 kb fingerprint regions. from low (red) to high (white). Compare with Figure S3. (TIF) [file pcbi.1002225.s004.tif]

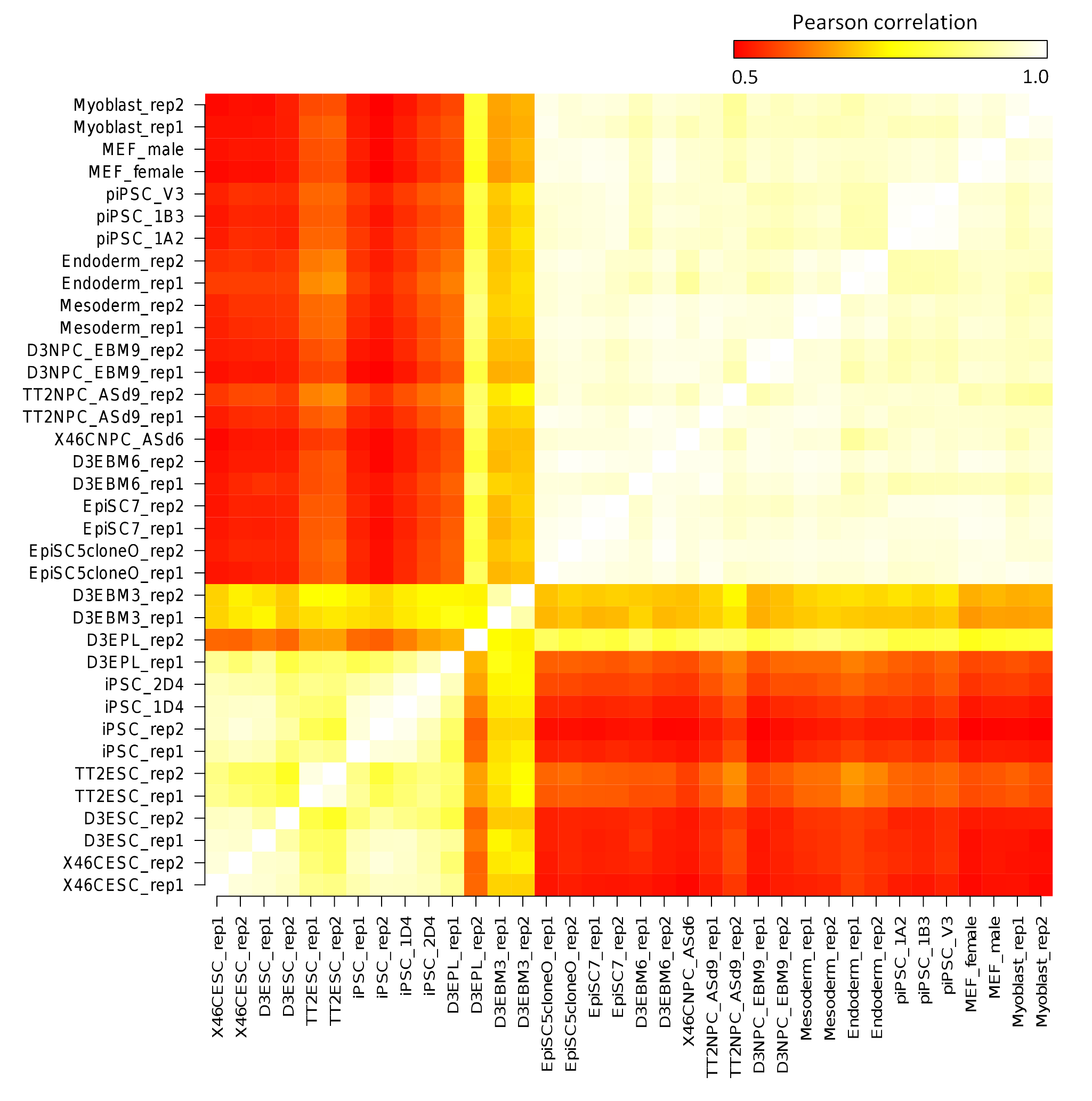

Supplement: Figure S5 — Correlations between mouse timing datasets in consensus pluripotency fingerprint regions. Heatmaps depict the level of correlation between timing datasets in 200 kb fingerprint regions, from low (red) to high (white). (TIF) [file pcbi.1002225.s005.tif]

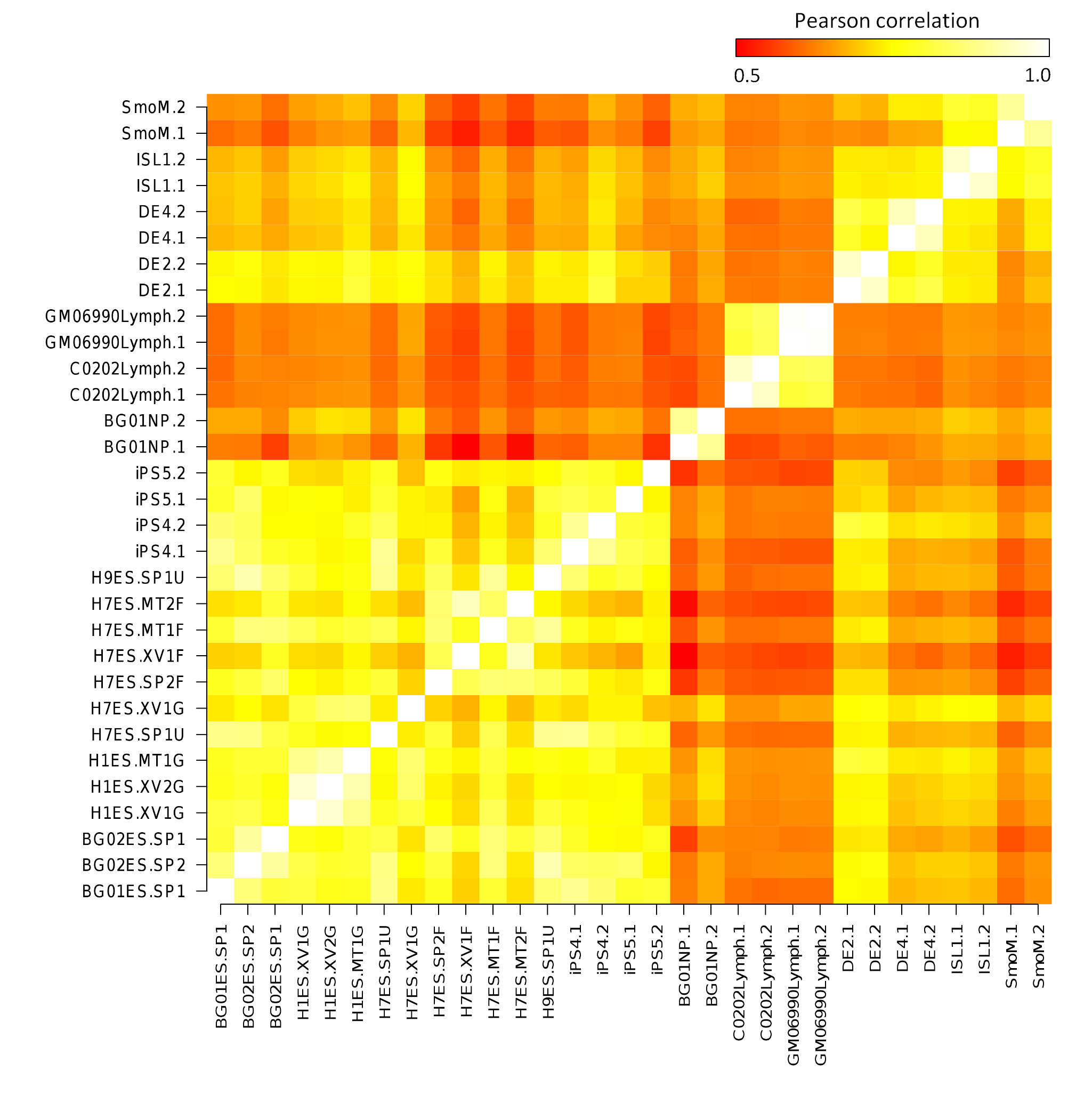

Supplement: Figure S6 — Genomewide correlations between human timing datasets. Heatmaps depict the level of correlation between timing datasets averaged in 200 kb windows, from low (red) to high (white). Note the relatively high level of variation in correlations between similar and divergent cell types (compare to Figure S7). (TIF) [file pcbi.1002225.s006.tif]

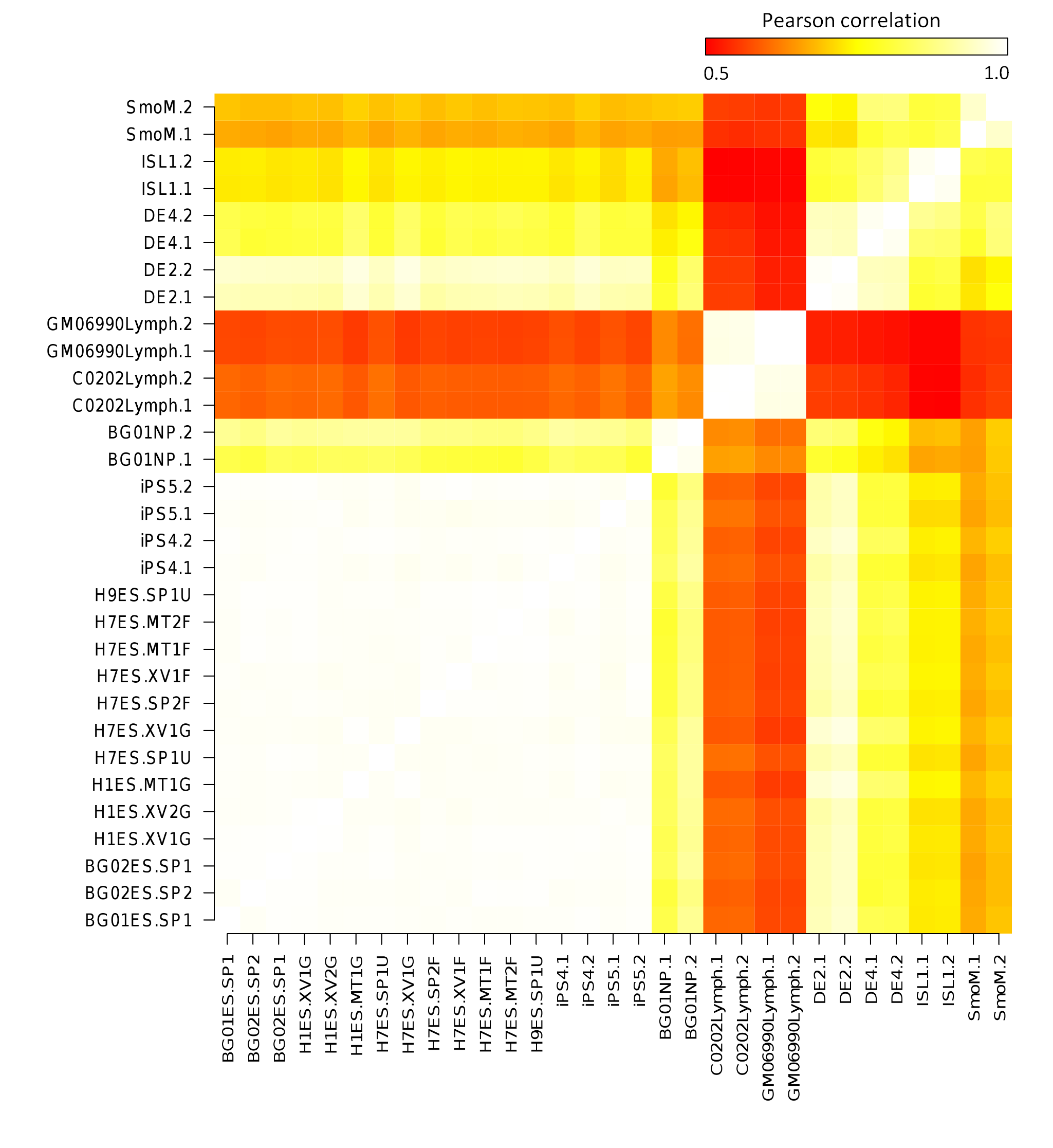

Supplement: Figure S7 — Correlations between human timing datasets in consensus cell-type fingerprint regions. Heatmaps depict the level of correlation between timing datasets in 200 kb fingerprint regions, from low (red) to high (white). (TIF) [file pcbi.1002225.s007.tif]

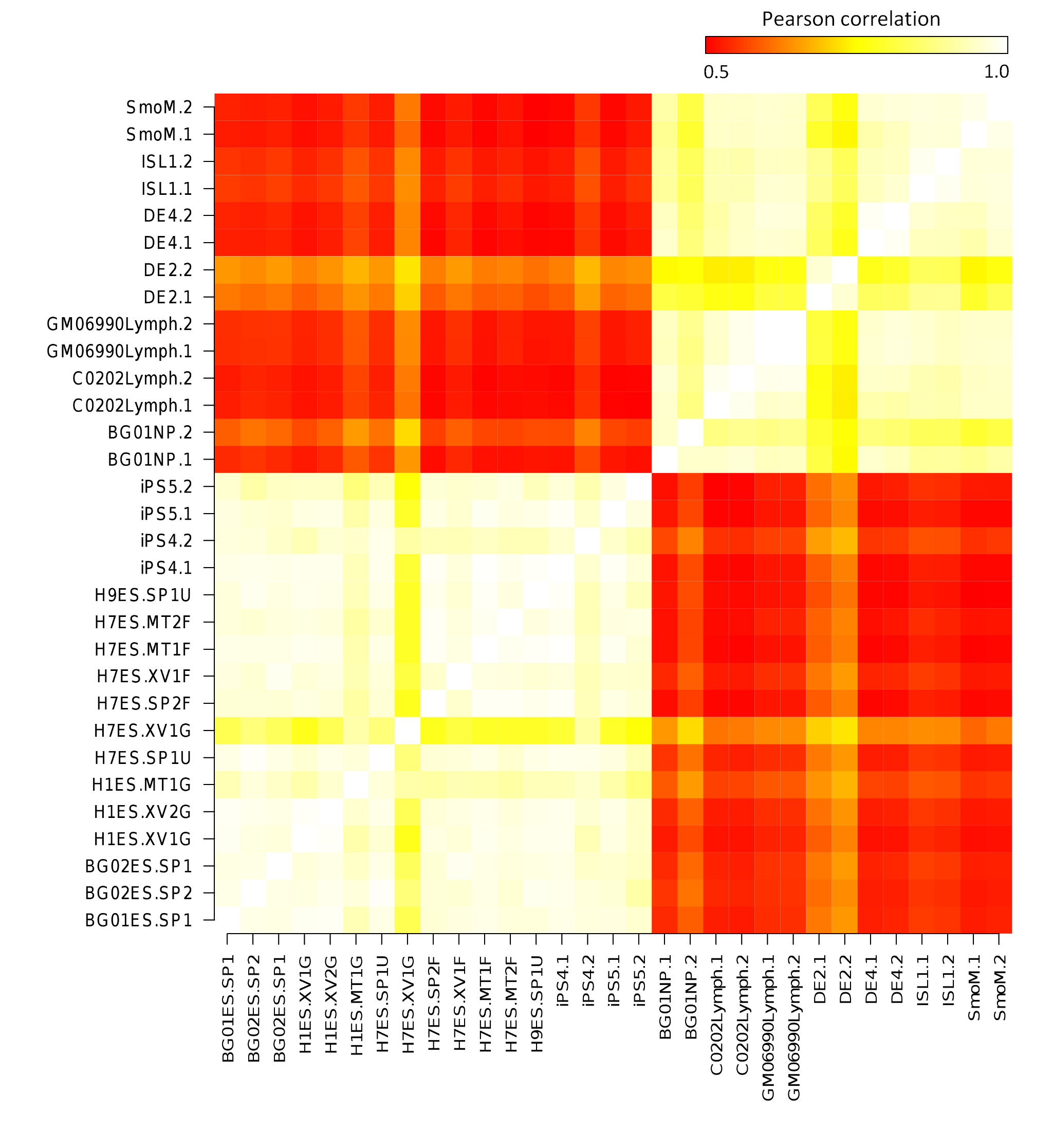

Supplement: Figure S8 — Correlations between human timing datasets in consensus pluripotency fingerprint regions. Heatmaps depict the level of correlation between timing datasets in 200 kb fingerprint regions, from low (red) to high (white). (TIF) [file pcbi.1002225.s008.tif]

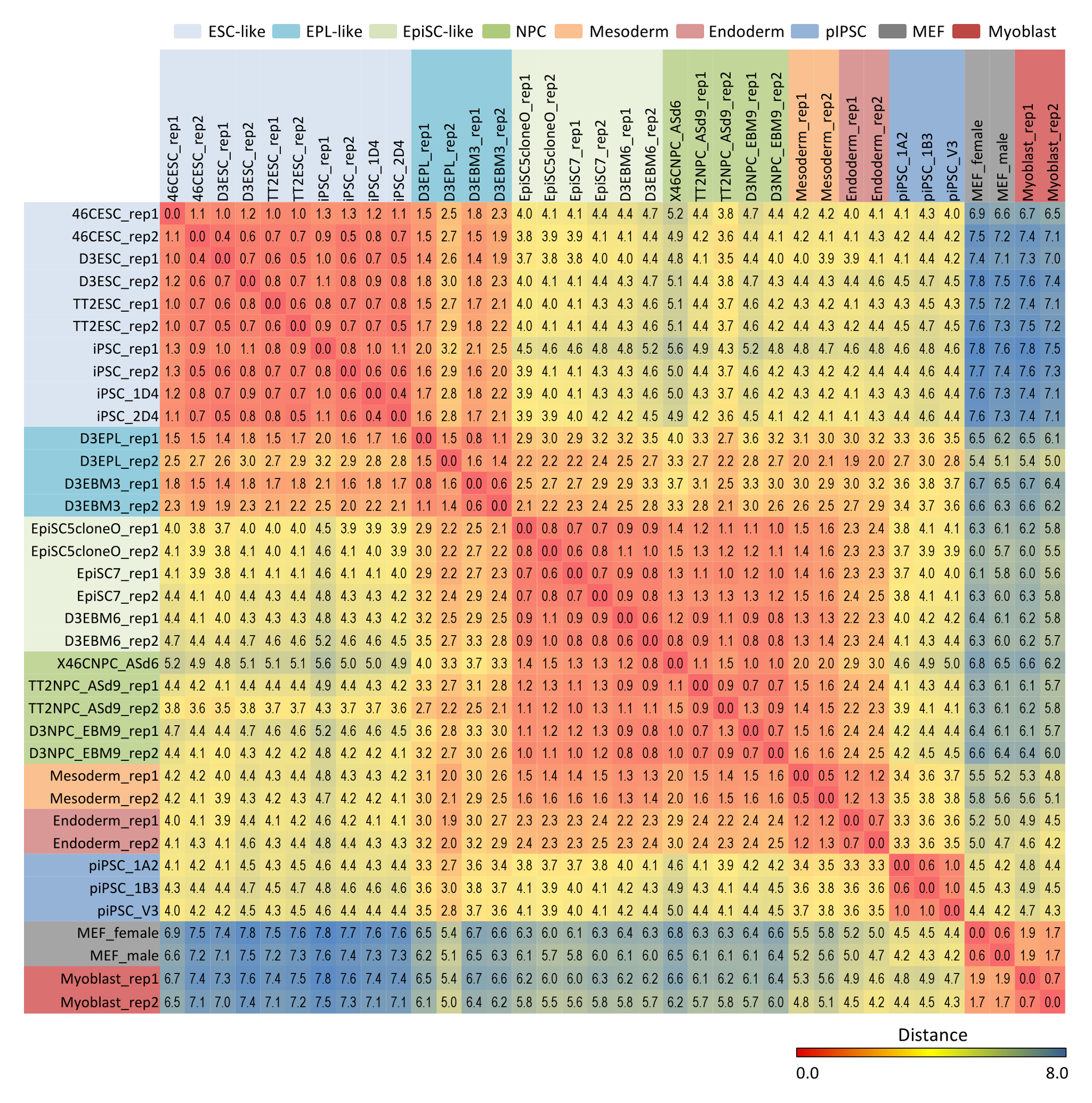

Supplement: Figure S9 — Distance matrix for mouse cell type consensus fingerprint. Numbers indicate the Euclidean distance between replication profiles measured in the 18 regions included in over 75% of runs of the fingerprinting algorithm. Cell type definitions used for training are indicated by the color map in rows and columns (see color key at top). Color scale for distances relates the relative similarity of cell types in fingerprint regions, from highly similar (red) to highly divergent (blue). (TIF) [file pcbi.1002225.s009.tif]

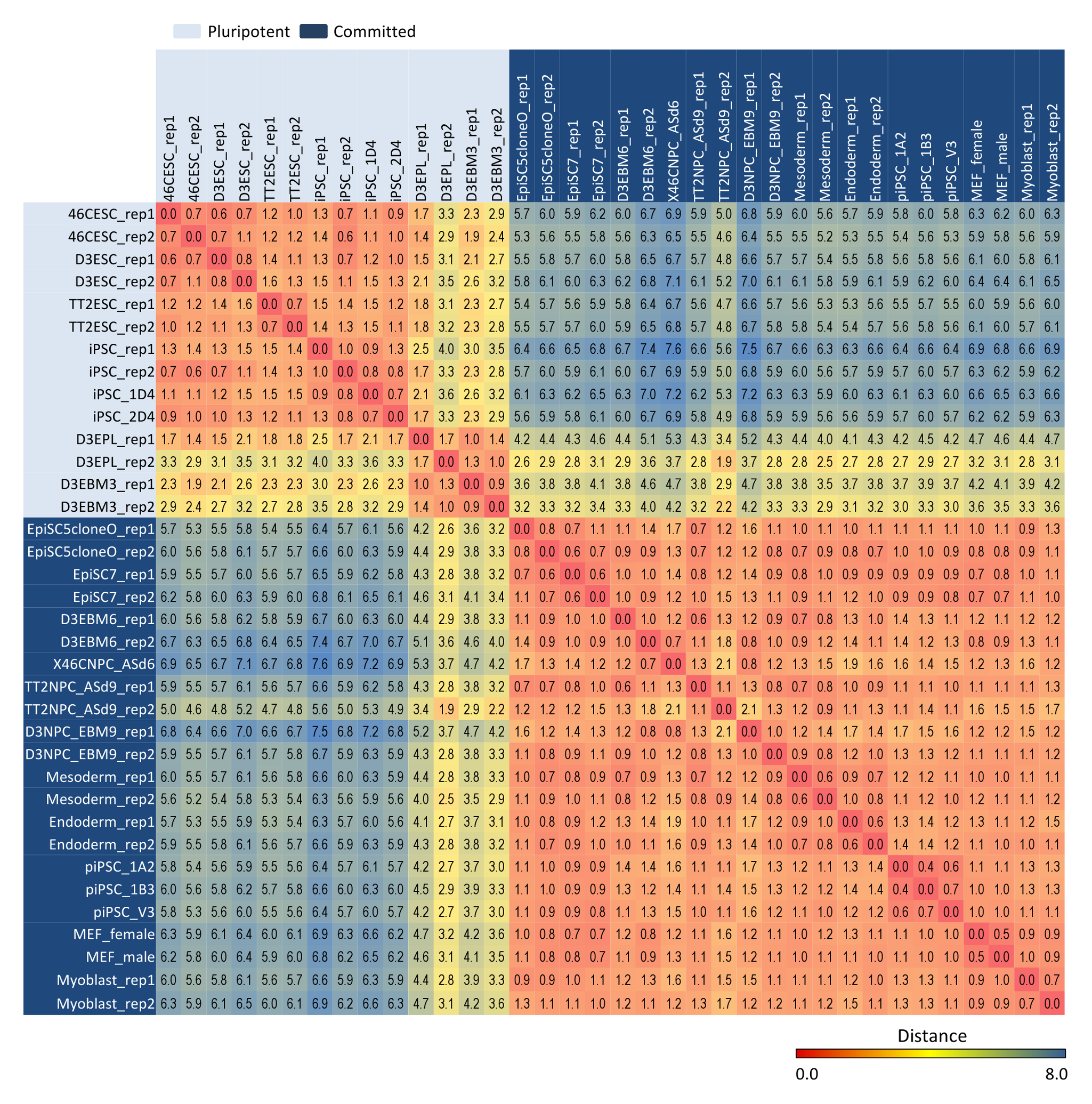

Supplement: Figure S10 — Distance matrix for mouse pluripotency consensus fingerprint. Numbers indicate the Euclidean distance between replication profiles measured in the 18 regions included in over 75% of runs of the fingerprinting algorithm. Cell type definitions used for training are indicated by the color map in rows and columns (light blue: pluripotent cell types; dark blue: committed cell types). Color scale for numbers relates the relative similarity of cell types in fingerprint regions, from highly similar (red) to highly divergent (blue). (TIF) [file pcbi.1002225.s010.tif]

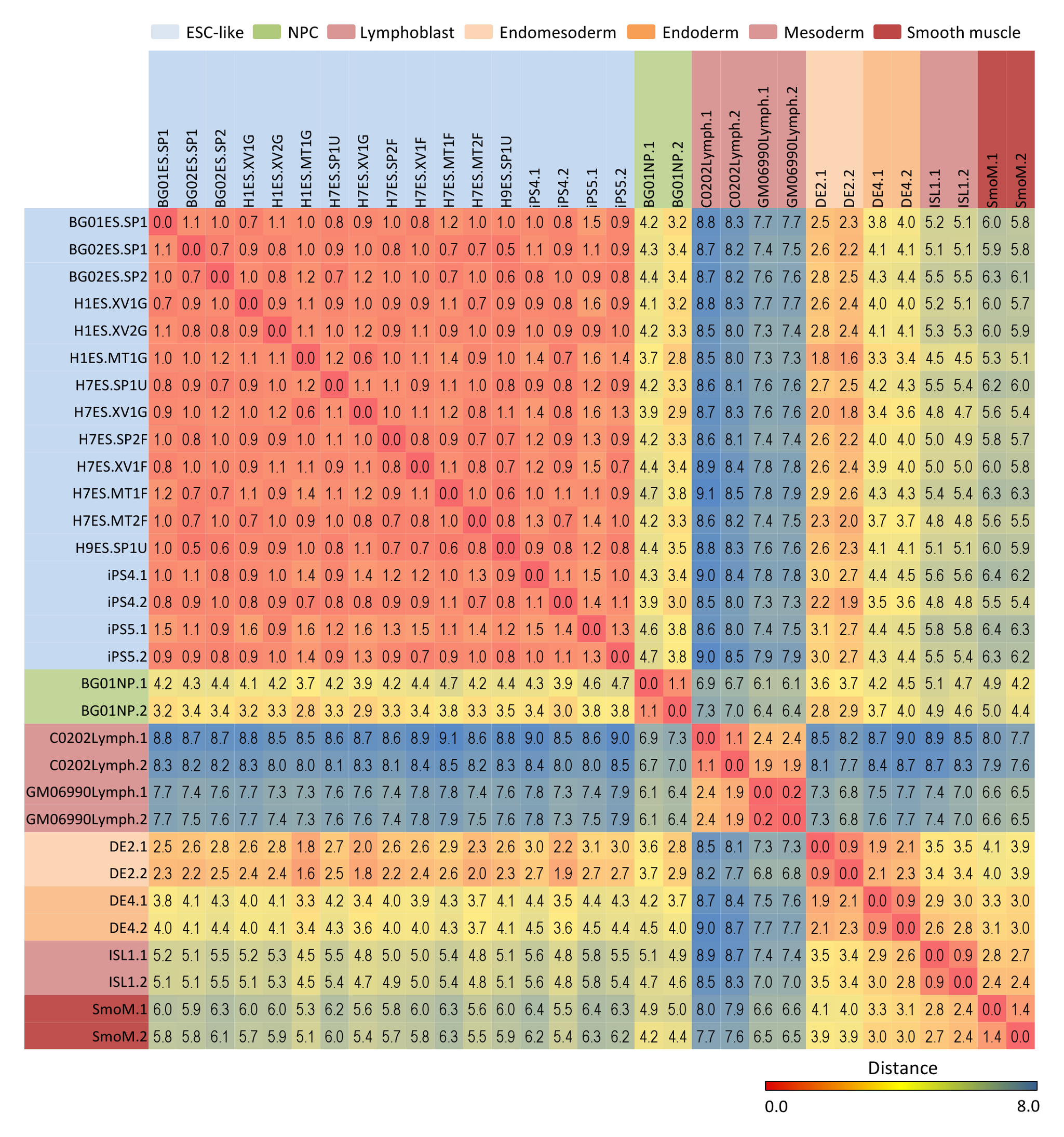

Supplement: Figure S11 — Distance matrix for human cell-type consensus fingerprint. Numbers indicate the Euclidean distance between replication profiles measured in the 18 regions included in over 75% of runs of the fingerprinting algorithm. Cell type definitions used for training are indicated by the color map in rows and columns (see color key at top). Color scale for numbers relates the relative similarity of cell types in fingerprint regions, from highly similar (red) to highly divergent (blue). (TIF) [file pcbi.1002225.s011.tif]

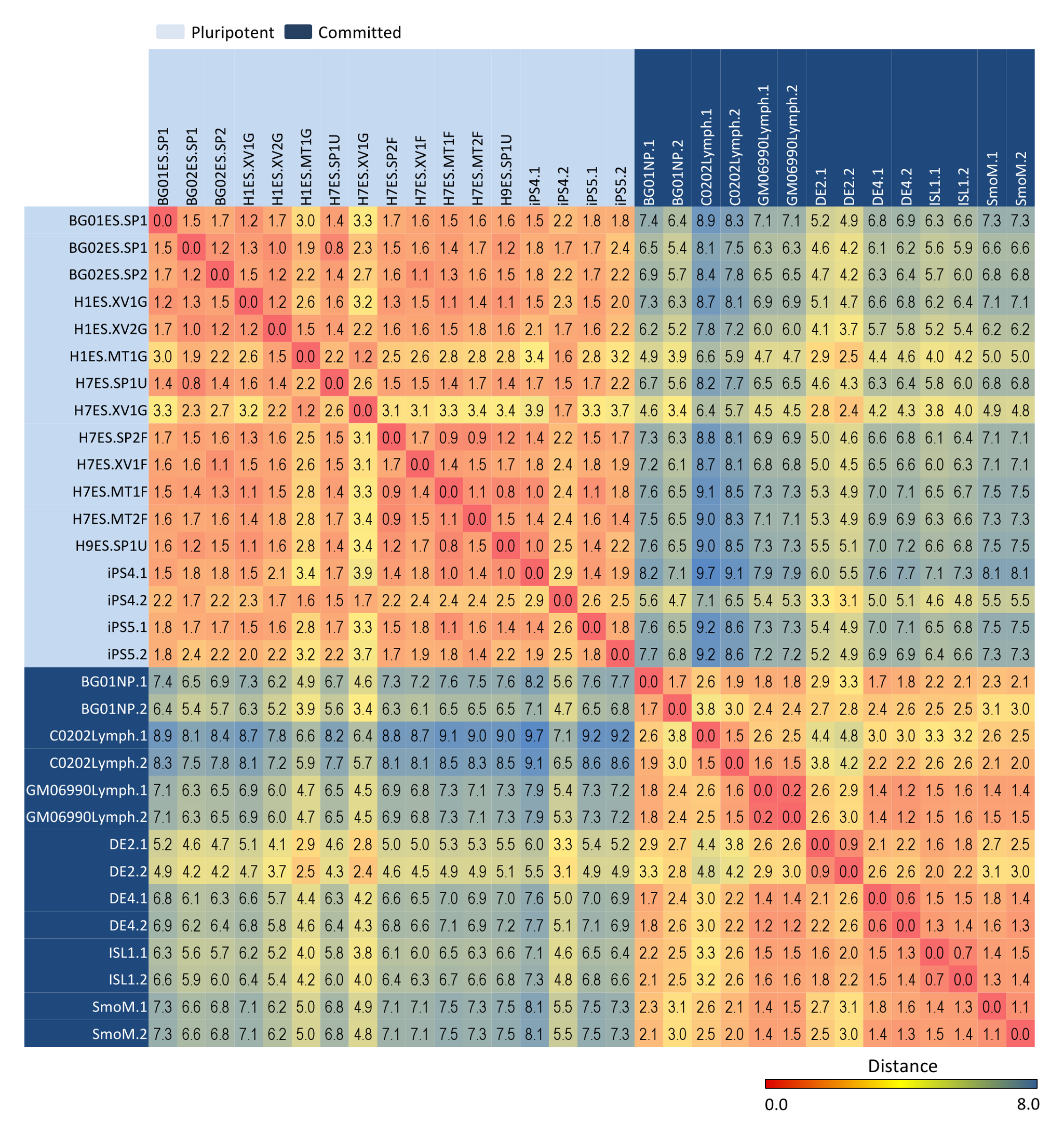

Supplement: Figure S12 — Distance matrix for human pluripotency consensus fingerprint. Numbers indicate the Euclidean distance between replication profiles measured in the 18 regions included in over 75% of runs of the fingerprinting algorithm. Cell type definitions used for training are indicated by the color map in rows and columns (light blue: pluripotent cell types; dark blue: committed cell types). Color scale for numbers relates the relative similarity of cell types in fingerprint regions, from highly similar (red) to highly divergent (blue). (TIF) [file pcbi.1002225.s012.tif]

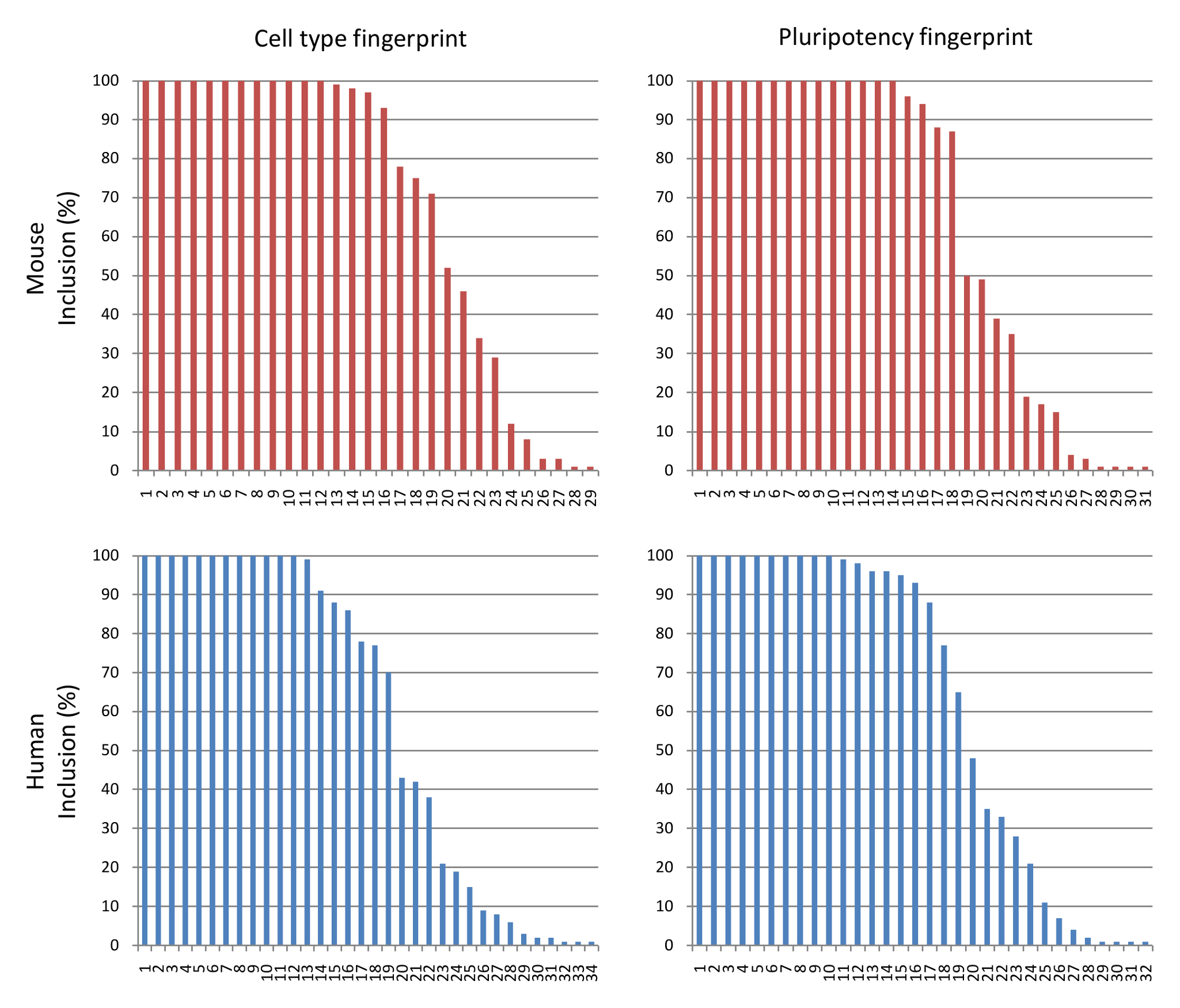

Supplement: Figure S13 — Calculation of consensus fingerprint regions. Since the Monte Carlo algorithm will randomly include or exclude regions in each run, the suitability of a set of regions for classification can be evaluated by running the algorithm multiple times and choosing the regions most often present. Regions with particularly unique timing in each cell type are often selected in 100/100 trials; here, we select regions included in at least 75 out of 100 runs for ‘consensus’ fingerprints for mouse and human cell type and pluripotency regions. The x-axis depicts the rank of each region in percentage of runs with that region included. (TIF) [file pcbi.1002225.s013.tif]
